# The role of pharmacists in eliminating counterfeit medicines in Nigeria

**DOI:** 10.3389/fpubh.2023.1170929

**Published:** 2023-08-22

**Authors:** Obi Peter Adigwe

**Affiliations:** Office of the Director General/Chief Executive Officer, National Institute for Pharmaceutical Research and Development, Abuja, Nigeria

**Keywords:** substandard, pharmaceuticals, healthcare, drugs, products, falsified

## Abstract

**Introduction:**

Over the years, counterfeit pharmaceuticals have posed immense concerns for global health and patient safety. This menace encompasses various classes of medications. Given the criticality of pharmacists’ interventions in drug distribution and supply, this study aimed at exploring their role in the prevention and control of counterfeit pharmaceutical products in Nigeria.

**Methods:**

A cross-sectional study was undertaken, using questionnaires to collect data from pharmacists across various sectors of pharmacy practice in Nigeria. Face and content validity was undertaken on the study tool prior to data collection. Ethical approval was obtained from the National Institute for Pharmaceutical Research and Development Health Research Ethics Committee, and confidentiality was strictly maintained during data collection process. Data were analyzed using Statistical Package for Social Sciences. Descriptive statistical analysis was undertaken and chi square was used to determine association between socio-demographic characteristics and variables.

**Results:**

The responses comprised 205 (52.6%) female and 185 (47.4%) male participants. Almost all the participants (98.4%) agreed that strict enforcement of drug laws can contribute to adequate control of counterfeit medicines in Nigeria, and majority of the study sample (64.7%) indicated that the poor implementation of these laws was a major factor influencing the preponderance of counterfeit medicines in the country. Two-thirds (63.5%) of the participants supported the need for pharmacists to provide adequate education to patients on strategies to identify counterfeit medicines, and a similar proportion (68.0%) were of the opinion that it was the responsibility of pharmacists to ensure that drugs are purchased from credible sources.

**Conclusion:**

Findings from this study, in addition to confirming pharmacists’ instrumentality in the fight against counterfeit medicines, identified certain context specific factors that can strengthen the regulation, policy and the entire healthcare system. Government and relevant stakeholders can therefore begin to articulate strategic reforms for contextual policy intervention that address medicines’ counterfeiting, whilst prioritising pharmacists’ role in other critical areas in the healthcare system.

## 1. Introduction

Medicines’ counterfeiting is a public health challenge affecting Nigeria and other Nations. The negative effect associated with this menace has significant deleterious implications on population’s health (1). According to White (2021), counterfeit medicines are of two categories, substandard and falsified (2). Substandard pharmaceuticals are products that fail to meet stipulated quality standards or specifications, whilst falsified drugs are deliberately or fraudulently produced to misrepresent their identity, source, or composition (3). Counterfeit medicines could therefore contain no active ingredient, wrong active ingredients, or ingredients at insufficient doses than is indicated on the label. On the other hand, they could be unapproved medicines, produced or repackaged to represent original brands (4).

Over the years, counterfeit pharmaceuticals have posed immense concerns for global health and patient safety, including drug resistance, treatment failure, and patient mortality (5). The threat posed by medicines’ counterfeiting is relevant to all types of therapeutic classes, especially with essential medicines such as anti-malarials and antibiotics (6). Worthy of focus, is the 280,000 sub-Saharan African children whose death have been linked with fake medications for pneumonia and malaria (7).

Since 1985, the problem of counterfeit medicines has been highlighted as a critical international priority by the World Health Organization (8). Efforts to successfully eradicate the menace have however proven rather difficult. Existing evidence suggests counterfeit medications still comprise a substantial percentage of the global market, with dollar estimates indicated at somewhere between US$200 billion and US$431 billion (9, 10). Unfortunately, Africa accounts for close to half of the global incidents of medicines’ counterfeiting (11). The predominant factors challenging access to quality medicines in Africa is attributable to a myriad of factors, including a high prevalence of unregulated drug markets; extremely porous pharmaceutical supply chain; and heavy reliance on drug importation (12, 13). According to the World Health Organization, these factors in addition to the poor public enlightenment on counterfeit drugs, and the erratic supply of medicines have largely encouraged the activities of counterfeiters (14).

In Nigeria, similar factors have been highlighted as limitations in the fight against counterfeit medicines (15, 16). This has prompted the government to take definite steps towards reducing the menace by undertaking relevant legislative processes aimed at regulating and controlling the manufacturing, sales, as well as distribution of drugs. In this regard, several laws such as the ‘Counterfeit, and Fake Drugs and Unwholesome Processed Foods (Miscellaneous Provisions) Act, Cap C.34 LFN 2004’ (17), the ‘Food and Drugs and Related products Act 2004’ (18), ‘National Agency for Food and Drug Administration and Control Decree No.15 of 1993’ (19), were enacted. These legislations represent a positive response by the country to forestall chaotic pharmaceutical distribution. It however appears from empirical data that the achievement of the underlying objectives of these laws against counterfeit medicines remain challenged in the country (20).

Internationally, pharmacist-led health system interventions have been identified as critical to successful counterfeit medicines reduction strategies (21–23). Central to these strategies are the professional roles pharmacists bring to bear in their engagement with health system actors, patients and the populace, in providing robust yet contextual mechanisms for limiting the circulation of counterfeit drugs and consequent health system challenges. In Nigeria however, these relationships and potential contributions to counterfeit medicines’ eradication remain largely unknown due to paucity of evidence in the contemporary literature. This study therefore aimed to better understand the status quo in the Nigerian setting, whilst providing critical insights on the roles of pharmacists in the mitigation of counterfeit pharmaceutical products.

## 2. Methods

A cross sectional study was undertaken between March and May 2022. A data collection tool (See Supplementary file) was developed following an extensive review of literature (21–24), the instrument was designed to assess roles of pharmacists in addressing medicines counterfeiting in Nigeria. The questionnaire was developed via an iterative process involving three faculty members with thematic research and teaching experience in the area of medicines’ counterfeiting. Each member of the team independently reviewed the questionnaire items and the suggested changes, additions, and deletions were effected. The revision process continued until a consensus was attained. The questionnaire items were eventually structured into four sections, to gain insights into the factors responsible for the circulation of counterfeit drugs as well as the strategies and the role of pharmacists towards mitigating the menace in Nigeria. This is in line with WHO global surveillance monitoring system which advocates keen awareness of the likely risk factors as critical in developing capacity to detect falsified medical products (3).

Face and content validations of the instrument were undertaken by an expert panel made up of three researchers that are pharmacists; the questionnaire was assessed for appropriateness, complexity, applicability, and attractiveness. Content validity was evaluated for each item of the questionnaire by a quantitative method. Content validity ratio and content validity index were tested for each item, and only those that passed these assessments were included in the final questionnaire. The questionnaire was pretested to ensure that its structure could assess pharmacists on the factors responsible for the circulation of counterfeit drugs as well as the strategies and the roles to be employed in the mitigation of the menace in Nigeria. The tool was pretested by administration to an initial cohort of 30 randomly selected participants. The feedback received did not necessitate any major change. Cronbach’s alpha test was also undertaken which gave a value of 0.862, indicating that the instrument was reliable.

The study was a national survey and a sample size of 377 was computed for a population of 20,000 pharmacists in Nigeria at 95% confidence level, 5% margin of error, and 50% response distribution using the Epi Info software version 7. Participants were selected using a stratified multistage sampling method. Recruitment was undertaken from at least one state across the six geopolitical zones in Nigeria to enable relevant and even assessment of pharmacists across the country. Participants were subsequently recruited randomly from the selected states.

The inclusion criteria for study participants were licensed pharmacists who were currently practicing in Nigeria with at least 1 year of experience and were willing to participate, as indicated by their informed consent. Participants who did not meet these requirements and those that provided completed questionnaires with many missing data were excluded from the study. Respondents were made up of pharmacists working in hospitals, administration, regulation, industries, non-governmental organizations, community, and importation.

Before the commencement of data collection, ethical approval was obtained from the Health Research Ethics Committee of the National Institute for Pharmaceutical Research and Development. Participation in the study was voluntary as informed consent was sought before the administration of the questionnaire. Data were collected using physical methods of questionnaire administration to pharmacists who had consented to the study and had signed the consent form. Absolute confidentiality was maintained during the data collection process.

The collected data were imported into Statistical Package for Social Sciences software (SPSS) version 25, after which descriptive statistical analysis was carried out and results were expressed in frequency and percentages. Inferential statistical analysis was also undertaken, and chi-square test was used to determine the association between variables. A value of *p* of 0.05 or less was considered the threshold for statistical significance.

## 3. Results

### 3.1. Demography and response rate

A total of 420 copies of questionnaires were administered and 390 valid responses were completed, giving a response rate of 92.9%. The participants comprised 205 (52.6%) females and 185 (47.4%) males. Slightly over a third of the sample (37.4%) had been in pharmacy practice for less than 5 years, whilst respondents who had been in practice for over 15 years constituted the least proportion (16.7%). Collectively, majority of the study participants (81.8%) practiced in hospital and community pharmacy settings, whereas, respondents practicing in the industrial sector represented the least proportion (0.5%) of the sample. Further details about socio-demographic characteristics are presented in Table 1.

**Table 1:** Socio-demographic characteristics.

Variable	Frequency (%)
Gender	
Male	185 (47.4)
Female	205 (52.6)
Age (years)	
≤30	166 (42.6)
31–40	125 (32.1)
41–50	37 (9.5)
Above 50	44 (11.3)
Missing data	18 (4.5)
Years of practice	
≤5	146 (37.4)
5–10	73 (18.7)
11–15	78 (20.0)
Above 15	65 (16.7)
Missing data	28 (7.2)
Highest level of education	
First degree	278 (71.3)
Master’s	95 (24.4)
PhD	4 (1.0)
Missing data	13 (3.3)
Area of practice	
Hospital	175 (44.9)
Community	144 (36.9)
Research/academia	13 (3.3)
Industry	2 (0.5)
NGO	14 (3.6)
Administration	17 (4.4)
Regulation	13 (3.3)
Missing data	12 (3.1)
FPCPharm	
Yes	45 (11.5)
No	293 (75.2)
Missing data	52 (13.3)

PhD, Doctor of Philosophy; NGO, Non-Governmental Organization; FPCPharm, Fellow of the Postgraduate College of Pharmacists.

### 3.2. Factors responsible for the circulation of counterfeit drugs

Despite the existence of laws against counterfeit medicines in Nigeria, about two-thirds of the study sample (64.7%) indicated that poor implementation of relevant policies and regulations hindered the fight against counterfeit medicines in the country. This sentiment was further affirmed by the strong majority (93.7%) that agreed that the existence of open drug markets contributed substantially to the prevalence of counterfeit medicines in the country. Other factors identified as responsible for the circulation of fake drugs include distribution challenges, lack of awareness and high dependence on importation. Further details are presented in Table 2.

**Table 2:** Factors responsible for the circulation of counterfeit drugs.

Statement	Strongly Disagree (%)	Disagree (%)	Neutral (%)	Agree (%)	Strongly Agree (%)
Inadequate enforcement of drug laws has facilitated the manufacture of counterfeit drugs in Nigeria	7 (1.8)	5 (1.3)	12 (3.1)	111 (29.1)	247 (64.7)
High cost of pharmaceuticals can increase the rate of medicines counterfeiting	13 (3.4)	34 (8.9)	40 (10.4)	162 (42.2)	135 (35.2)
Drug distribution by non-pharmacists can contribute to the spread of counterfeit drugs	7 (1.8)	9 (2.4)	21 (5.5)	117 (30.6)	228 (59.7)
Inadequate public awareness can contribute to the prevalence of drug counterfeiting in the health sector	7 (1.8)	10 (2.6)	8 (2.1)	140 (36.6)	218 (56.9)
High dependence on importation of pharmaceutical products is a substantial cause of counterfeit medicines’ circulation	11 (2.9)	41 (10.7)	52 (13.5)	150 (39.1)	130 (33.9)
Fake medicines are prevalent due to drug purchase from compromised sources	6 (1.6)	14 (3.7)	11 (2.9)	140 (36.6)	212 (55.4)
The existence of unregulated open drug market in Nigeria has contributed substantially to the prevalence of fake drugs	8 (2.1)	5 (1.3)	11 (2.9)	88 (22.9)	272 (70.8)

### 3.3. Strategies to reduce the circulation of counterfeit medicines

With respect to the factors contributing to the circulation of counterfeit medicines in Nigeria, Table 3 highlights several strategies aimed at a more proactive engagement with the issue. Almost all the participants (98.4%) responded that stricter drug laws can enable the control of counterfeit medicines in Nigeria. Similarly, preemptive formulation and implementation of policies and regulations that control online drug commerce and importation was advocated by a majority of the sample (95.3%). Furthermore, about three-quarters (72.3%) of the study participants indicated that regular training for pharmacists was necessary to enhance the capacity to detect counterfeit pharmaceuticals. A strong majority (92.9%) also acknowledged that the use of drug testing and screening technologies would enhance the detection of fake drugs in Nigeria.

**Table 3:** Strategies to reduce the circulation of counterfeit medicines.

Statement	Strongly Disagree (%)	Disagree (%)	Neutral (%)	Agree (%)	Strongly Agree (%)
It is important to organise regular training for pharmacists in the aspect of detecting counterfeit medicines	4 (1.0)	2 (0.5)	11 (2.9)	89 (23.2)	277 (72.3)
Restriction of persons who are not pharmacists from acting as a pharmaceutical sales representative can reduce counterfeit medicines distribution	3 (0.8)	25 (6.5)	46 (12.0)	112 (29.3)	196 (51.3)
Implementing new drug testing technologies in the country to screen for drug authenticity such as the Radio Frequency Identification (RFID) will reduce the spread of counterfeit products	2 (0.5)	3 (0.8)	22 (5.7)	110 (28.7)	246 (64.2)
Development of local pharmaceutical industry is key to reduce import of fake medicines	3 (0.8)	16 (4.3)	34 (9.0)	120 (31.9)	203 (54.0)
A more stringent law that discourages import of counterfeit pharmaceutical products should be enacted	1 (0.3)	10 (2.6)	16 (4.2)	107 (28.2)	246 (64.7)
There is need to strengthen regulatory activities in all aspect of pharmaceutical supply chain	1 (0.3)	2 (0.5)	10 (2.6)	100 (26.3)	267 (70.3)
It is important for pharmacy regulatory agencies to ensure proper implementation and strict enforcement of drug laws	1 (0.3)	1 (0.3)	4 (1.1)	119 (31.4)	254 (67.0)
It is critical for government agencies to inculcate policies that can address and monitor online sale of medicines	1 (0.3)	4 (1.1)	13 (3.4)	122 (32.2)	239 (63.1)

### 3.4. Pharmacists’ roles in mitigating counterfeit medicines’ circulation

Pharmacists are drug experts whose proficiency in medicines and healthcare can effectively help to address counterfeit medicines circulation across different health settings in Nigeria. This section highlights various responsibilities of pharmacists towards addressing counterfeit drugs in the country.

#### 3.4.1. Patient-facing roles

**Figure 1:**
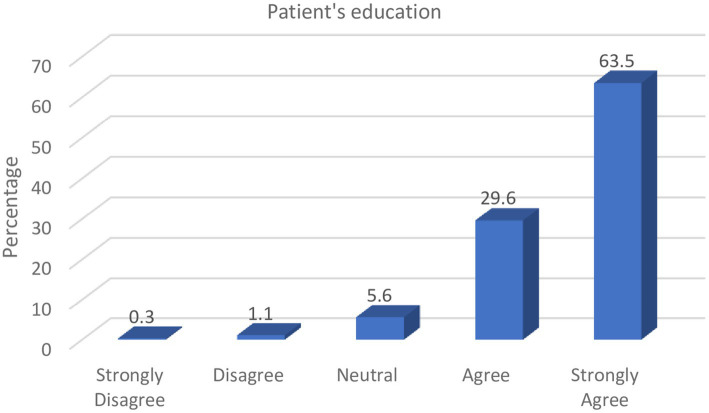
Role of pharmacists in patient education.

Patients’ awareness of counterfeit medicines can reasonably limit its purchase, use, and consequent effect on health. From Figure 1, it can be observed that pharmacists consider it their responsibility to discourage patients from the purchase of counterfeit medicines by educating them on the dangers of such drugs and how they can be identified.

Majority of the respondents (93.1%) indicated that pharmacists can play critical roles in educating patients on the various consumer verification strategies to examine the authenticity of medicines at the point-of-purchase, as well as discourage them from obtaining medicines from unreliable drug sources.

#### 3.4.2. Roles in procurement

**Figure 2:**
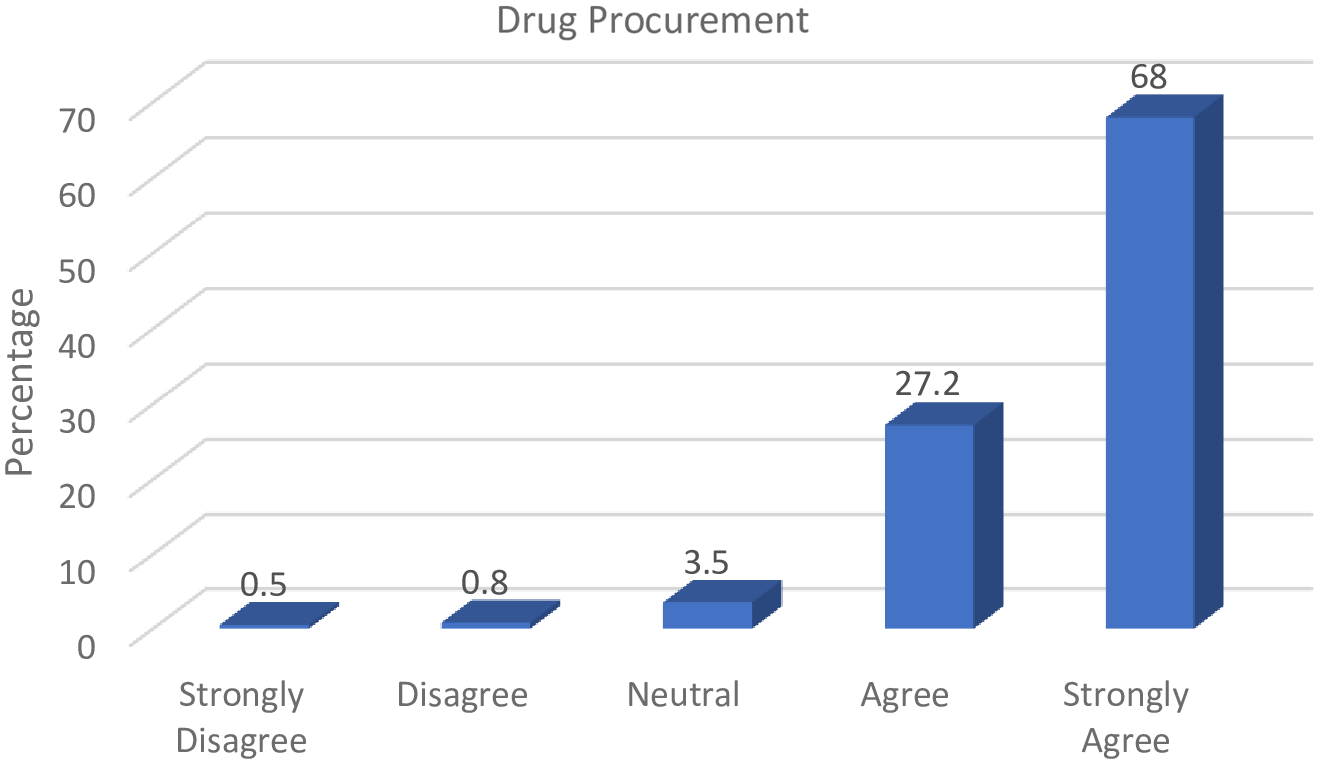
Role of pharmacists in drug procurement.

Pharmacists also provide specialized services in the health sector with regards to drug information, as well as planning and monitoring of drug programmes for health improvement. These professionals can therefore play critical roles in drug procurement, especially given their knowledge of medication brands and sources. The responses from Figure 2 illustrate respondents’ perceptions regarding the impact on the circulation of counterfeit medicines in the country.

Over two-thirds of the study participants (68.0%) strongly affirmed that it was the responsibility of pharmacists to verify drug procurement sources as well as to ensure that the available pharmaceuticals within their various areas of practice were obtained from certified drug sources such as manufacturers and licensed drug distributors.

#### 3.4.3. Roles in drug assessment and reports

**Figure 3:**
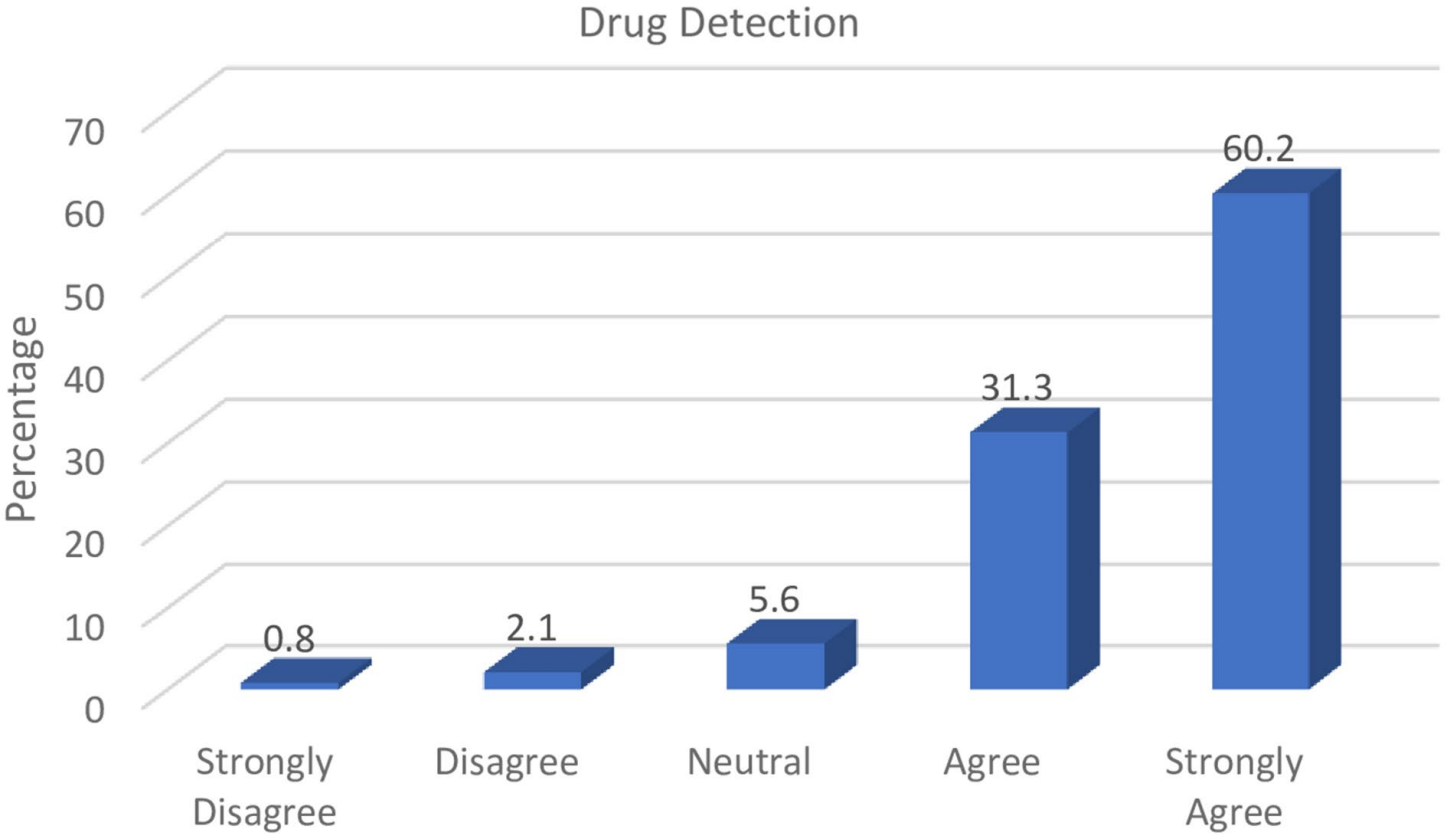
Role of pharmacists in drug assessment.

To a trained professional such as a pharmacist, several factors can enable the identification of counterfeit medicines. These include unrealistic expiry dates, misspellings on drug leaflets, inconsistencies in packaging, absence of seals/embossments, or even patient adverse reaction reports. The results represented in Figure 3 indicate that drug assessments through visual scrutiny is an important aspect of counterfeit medicines detection that should be adopted by pharmacists.

The majority of respondents (91.50%) in the study indicated that it was the responsibility of pharmacists to differentiate genuine from counterfeit drugs given their proficiency in detecting counterfeit medicines through visual scrutiny and investigation of adverse patient reports.

**Figure 4:**
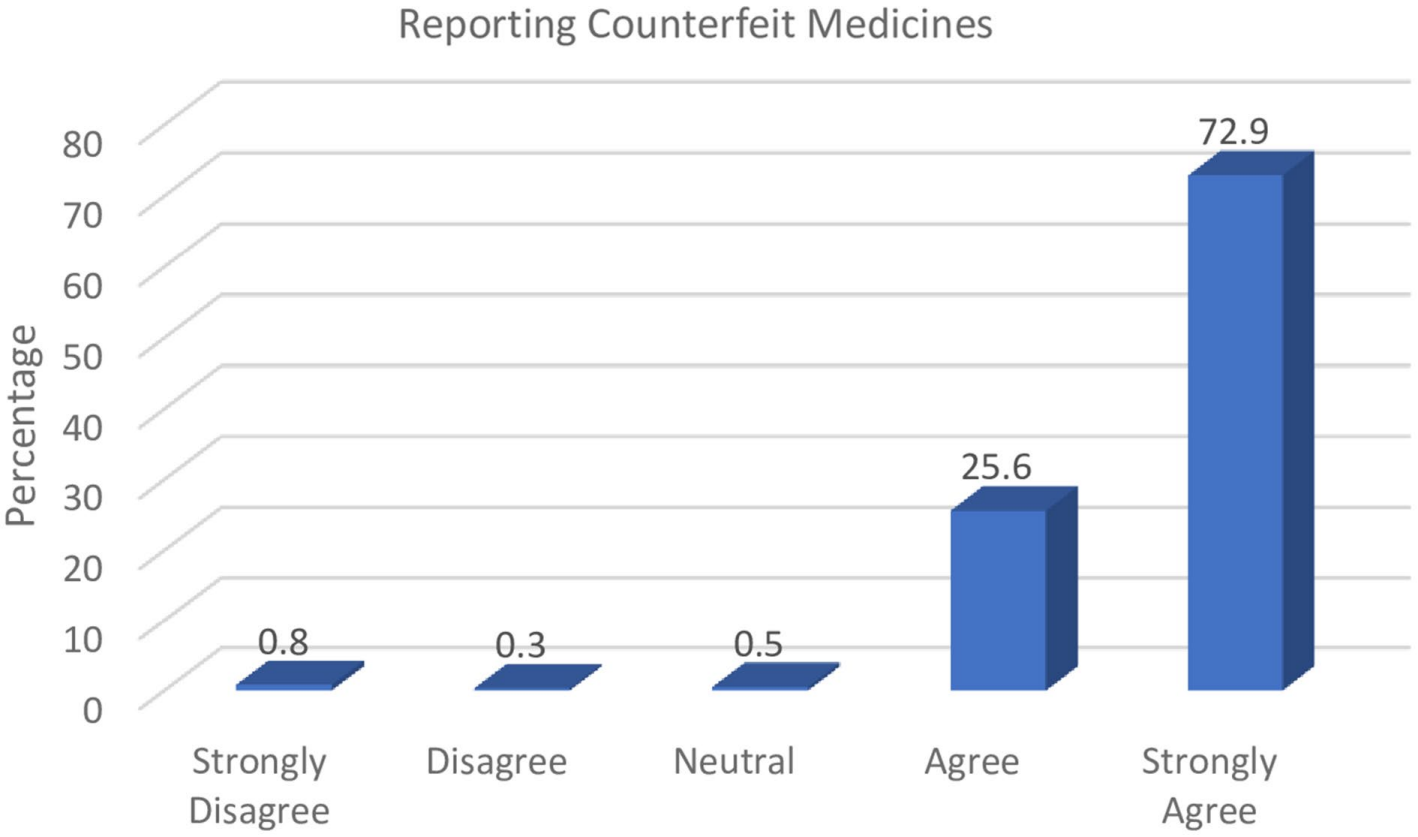
Role of pharmacists in reporting counterfeit drugs.

Furthermore, findings in Figure 4 revealed that the study participants were of the opinion that upon suspicion of counterfeit drugs, pharmacists ought to live up to their responsibility of reporting such events to the regulatory authorities.

Almost all respondents in this study (98.5%) acknowledged the importance and responsibility of pharmacists in reporting suspected counterfeit medicines to the drug authorities rather than discarding them.

### 3.5. Association between variables and demography

Findings from the inferential statistical analysis undertaken revealed that some of the responses of the study participants were influenced by their socio demographic characteristics. More of the participants who are above 50 years of age indicated that high dependence on importation of medicines contributed substantially to the rate of faking and counterfeiting in Nigeria as compared to the younger pharmacists. Similarly, it also emerged that more of the participants with longer years of practice experience were of the view that importation of pharmaceutical products into the country was one of the factors responsible for the proliferation of counterfeits in the country. Relevant details are provided in Table 4.

**Table 4:** Cross-tabulation of demography with high dependence on importation of pharmaceutical products as a substantial cause of counterfeit medicines in circulation.

Demography	Strongly Disagree (%)	Disagree (%)	Neutral (%)	Agree (%)	Strongly Agree (%)	*X* ^2^	Value of *p*
Age (Years)							
≤30	1 (0.6)	17 (10.5)	31 (19.1)	68 (42.0)	45 (27.8)	39.447	<0.001
31–40	2 (1.6)	15 (12.2)	16 (13.0)	41 (33.3)	49 (39.8)		
41–50	5 (13.9)	5 (13.9)	5 (13.9)	12 (33.3)	9 (25.0)		
Above 50	3 (6.7)	2 (4.4)	0 (0.0)	24 (53.3)	16 (35.6)		
Years of practice							
≤5	0 (0.0)	10 (7.0)	26 (18.2)	61 (42.7)	46 (32.1)	29.857	0.003
5–10	1 (1.4)	12 (16.4)	11 (15.1)	23 (31.5)	26 (35.6)		
10–15	4 (5.2)	12 (15.8)	10 (13.2)	27 (35.5)	23 (30.3)		
Above 15	5 (7.6)	4 (6.1)	2 (3.0)	29 (43.9)	26 (39.4)		

## 4. Discussion

This study has provided novel insights regarding contextual strategies that enable pharmacists’ professional roles contribute to addressing medicines’ counterfeiting in Nigerian setting. Findings that emerged from this study suggest that despite the existence of laws against counterfeit and fake medicines in Nigeria, ineffectual legislation and suboptimal enforcement remain critical challenges. This is similar to previous findings reported over two decades ago (24) and therefore suggests a lack of attention to this crucial area of healthcare. This situation requires contextual, yet urgent intervention, considering the fact that several laws such as Counterfeit and Fake Drugs Act have been in existence for decades to regulate medicines’ counterfeiting. Poor implementation of some of these laws seems to constitute a major barrier to the eradication of counterfeit pharmaceuticals in Nigeria. Stricter laws alongside more robust implementation will reduce the prevalence of this burden that is of public health concern. The existence of open-drug markets was identified as a major factor substantially contributing to the proliferation of counterfeit medicines in Nigeria, and this is in line with a previous report (25). Given the risk posed by open-drug markets with respect to the infiltration of counterfeit drugs in the pharmaceutical supply chain, it is important that licensed pharmacists are mandated to control all aspects of the supply chain. Professional responsibility for these critical aspects of the chain would enable pharmacists deploy their expertise in drug procurement, distribution and assessment, towards preventing subversion by fakes and counterfeits.

Almost all the respondents agreed that pharmacists have the responsibility to verify and purchase medications from certified drug sources. This finding buttresses the fact that despite the porosity of drug supply chain and the chaotic drug market in Nigeria (26), the role of pharmacists in drug procurement can substantially contribute to addressing counterfeit medicines’ circulation. This strategy was similarly highlighted by pharmacists in other setting, and this led to a reduction in consumption of counterfeit medicines by patients (27). Pharmacists are healthcare professionals equipped with knowledge of medicines, and can therefore harness their proficiencies in this regard to ensure genuine products are purchased from accredited distributors or manufacturers. Despite the utility of these professionals in the fight against counterfeiting, emerging evidence suggests an increasing shortage of pharmacists in the country, especially within private hospitals where there are few or no pharmacists to handle procurement activities (28, 29). This may pose a challenge to a contextual adoption of this strategy in addressing faking and counterfeiting. In recent times, it has been reported that the pharmacists-to-patient ratio in the country is at one pharmacist to 14,000 persons which is a contravention of the WHO recommendation of one pharmacist to 2,000 patients (30). The consequent workload on pharmacists within healthcare facilities has a tendency to therefore reduce activities in this critical area. Enhancing the pharmacy workforce in the health sector is therefore critical in the overarching strategy aimed at reducing the circulation of counterfeit drugs in Nigeria.

More than two-thirds of the participants in this study indicated that high cost of medicines can precipitate unwilling purchase of cheaper and potentially counterfeit alternatives. Suboptimal efforts to enable the prioritization of medicines security over time, means that local manufacturing of drugs and commodities have sometimes been subordinated in favour of imported analogues. Incidentally, regulatory oversight disparities between foreign and local factories have been associated with importation of fakes and counterfeits (31, 32). To bridge this gap, the participants further indicated that in an event, where the cost of drugs are unaffordable, it is the responsibility of pharmacists to educate the drug procurement team, and patients on the most appropriate generic alternatives, given their knowledge on pharmaco-economics. This is of critical importance and can help reduce patients’ exposure to counterfeit drugs. This is especially relevant for programmes such as the National Health Insurance Scheme where generic alternatives constitute the bulk of procured medications.

This study identified the need for pharmacists to take up the responsibility of educating patients on the dangers and risks of counterfeit drugs. This finding is essential given that poor public awareness of drug counterfeiting and its consequent effects predisposes the patient population to vulnerabilities associated with use of fake medicines. This aligns with findings by Alfadl et al., (33) which linked poor consumer awareness with unprecedented purchase of counterfeit medicines in the study setting (33). The emergent findings from this study further highlights specifics for policy and practice implementation, for instance the exhortation for patients to desist from patronizing unlicensed premises. Participants supported the need for government to develop contextual policies to regulate and control online sales of pharmaceutical products. As previously reported by Liang (2006), internet drug commerce poses considerable risks for the infiltration of counterfeit medicines into the pharmaceutical supply chain (34).

Almost all the participants were of the view that it was important for pharmacists to promptly report suspicious pharmaceutical products to the regulatory authorities, suggesting a strong sense of responsibility that aligns with professional ethics for pharmacists. This finding can support the development of tools and frameworks that facilitate comprehensive monitoring of relevant supply chains, as well as capacity development modules that enable robust engagement in the field. Pharmacists are already able to identify fake and counterfeit medicines through techniques such as seals and embossments; holograms; and consistency of expiry dates (35). Given the progressive sophistication in criminal drug counterfeiting, it is imperative for pharmacists to continually update their knowledge and skills with respect to identification and validation techniques. This has been similarly recommended by the American Pharmacists Association in addressing this menace in the United States (27). Furthermore, participants in this study supported the need to adopt relevant technologies such as the Radio Frequency Identification (RFID) to curb medicines’ counterfeiting. RFID technology can enhance the efficiency in pharmaceutical supply chain and reduce the prevalence of counterfeits in the system.

Finally, findings from this study revealed that older pharmacists as well as those with longer years of practice experience were of the view that a high dependence on importation contributed to the menace of counterfeit medicines’ circulation in Nigeria. Considering that this standpoint was from those with more understanding of the pharmaceutical value chain as a result of their longer experience, it is of critical importance that government consider this perspective in policy reforms aimed at eliminating medicines’ counterfeiting. These emergent findings further buttress the need to prioritize local production of medicines, so as to better guarantee access to high quality products in Nigeria (31).

Although the findings of the study have provided new insights in the study area, limitations exist due to the positivist research methodology adopted. Further studies following a qualitative methodology can enable an in dept exploration of relevant strategies that will underpin successful implementation of laws that will counter medicines counterfeiting.

## 5. Conclusion

Drug counterfeiting has become a multi-billion-dollar industry threatening public health and threatening public trust in the healthcare system. This study revealed factors such as poor legislation, inadequate public awareness and porosity of the drug supply chain as serious challenges to the eradication of counterfeit medicines in the country. It is therefore important that the government adopt critical strategies and coordinated anti-counterfeiting initiatives, underpinned by the findings from this study to address the infiltration of fake medicines in the healthcare system.

Findings from this study revealed that pharmacists’ professional training, knowledge and skills can contribute significantly to the fight against medicines’ counterfeiting. In developing and implementing anti-counterfeiting policies, it is important for the government to adopt a comprehensive multidisciplinary approach that robustly engages this professional group, alongside other relevant stakeholders within and outside the health sector. This will ensure that policies and practices that emanate from the process are sustainable and sufficiently contextual to the setting.

Based on the novel findings from this study, there is an urgent need to prioritize policy and practice reforms in legislation, regulation, enforcement, capacity building, and public enlightenment. Initiatives that encourage local manufacturing of medicines are critical as a means of assuring Medicines’ Security. This will in turn support the elimination of system gaps that facilitate infiltration of counterfeit medicines through importation. Further studies are however required to deepen these emergent findings to better understand subpopulation disparities in perceptions around faking and counterfeiting, and how these can influence policy and practice.

## Data availability statement

The raw data supporting the conclusions of this article will be made available by the author, without undue reservation.

## Ethics statement

The study protocol was reviewed and approved by National Institute for Pharmaceutical Research and Development Health Research Ethics Committee. The participants provided their written informed consent to participate in this study.

## Author contributions

The author confirms being the sole contributor of this work and has approved it for publication.

## Conflict of interest

The author declares that the research was conducted in the absence of any commercial or financial relationships that could be construed as a potential conflict of interest.

## Publisher’s note

All claims expressed in this article are solely those of the authors and do not necessarily represent those of their affiliated organizations, or those of the publisher, the editors and the reviewers. Any product that may be evaluated in this article, or claim that may be made by its manufacturer, is not guaranteed or endorsed by the publisher.
