# Supplementary material for: The role of pharmacists in eliminating counterfeit medicines in Nigeria
Source: Front Public Health. 2023 Aug 22;11:1170929. doi: 10.3389/fpubh.2023.1170929 (PMC10477360; doi:10.3389/fpubh.2023.1170929)
Supplement: Supplementary file 1 [file Table_1.DOCX]

**QUESTIONNAIRE**

**The Role of Pharmacists in Eliminating Counterfeit Medicines in Nigeria**

**Introduction**

Counterfeit and fake drugs have become a serious menace trending across Nigeria with critical health consequences. The questionnaire aims to assess the views of pharmacists with regards factors responsible for the circulation of counterfeit drugs as well as their role in preventing fake medicines circulation. Please fill the questionnaire by ticking (√) the most appropriate option (s). Your responses will be anonymised and treated confidentially.

**Section A: Demographic data**

| Male | Female |
| --- | --- |

1. **Gender**
2. **Age**

| >30years | 31-40years | 41-50years | Above 50years |
| --- | --- | --- | --- |

1. **Years of practice**

| <5years | 5-10 | 11-15years | Above 15years |
| --- | --- | --- | --- |

1. **Area of practice**

| Hospital | Community | Research/Academia | Industry | Others, please specify ....................................... |
| --- | --- | --- | --- | --- |
| NGO | Importation | Administration | Regulation |  |

| First degree | Masters | PhD |
| --- | --- | --- |

1. **Highest level of education**

For the following sections (B - D), please give your rating: **SD (Strongly Disagree), D (Disagree), N (Neutral), A (Agree), SA (Strongly Agree)**

**Section B: Factors responsible for the circulation of counterfeit/fake drugs**

| 1. **SN** | 1. **Statement** | 1. **SD** | 1. **D** | 1. **N** | 1. **A** | 1. **SA** |
| --- | --- | --- | --- | --- | --- | --- |
| 1. 1 | 1. Inadequate enforcement of drugs laws has facilitated the manufacture of fake/counterfeit drugs in Nigeria |  |  |  |  |  |
| 1. 2 | 1. High cost of pharmaceuticals can increase the rate of medicines counterfeiting |  |  |  |  |  |
| 1. 3 | 1. Drug distribution by non-pharmacists can contribute to the spread of counterfeit/fake drugs |  |  |  |  |  |
| 1. 4 | 1. Inadequate public awareness can contribute to the prevalence of drug counterfeiting in the health sector |  |  |  |  |  |
| 1. 5 | 1. High dependence on importation of pharmaceutical products is a substantial cause of counterfeit medicines in circulation. |  |  |  |  |  |
| 1. 6 | 1. Fake medicines are prevalent due to drug purchase from compromised sources |  |  |  |  |  |
| 1. 7 | 1. The existence of open drug market in Nigeria has contributed substantially to the prevalence of fake drugs. |  |  |  |  |  |

**Section C: Strategies to reduce the circulation of fake/counterfeit drugs**

| **S/N** | **Statement** | **SD** | **D** | **N** | **A** | **SA** |
| --- | --- | --- | --- | --- | --- | --- |
| 1. 1. | It is important to organise regular training for pharmacists in the aspect of detecting counterfeit medicines. |  |  |  |  |  |
| 1. 2. | Restriction of persons who are not pharmacists from acting as a pharmaceutical sales representative can reduce counterfeit medicines distribution. |  |  |  |  |  |
| 1. 3. | Implementing new drug testing technologies in the country to screen for drug authenticity such as the Radio Frequency Identification (RFID) will reduce the spread of counterfeit products |  |  |  |  |  |
| 1. 4. | Development of local pharmaceutical industry is key to reduce import of fake medicines |  |  |  |  |  |
| 1. 6. | A more stringent law that discourages import of counterfeit pharmaceutical products should be enacted |  |  |  |  |  |
| 1. 7. | There is need to strengthen regulatory activities in all aspect of pharmaceutical supply chain |  |  |  |  |  |
| 1. 8. | It is important for pharmacy regulatory agencies to ensure proper implementation and strict enforcement of drug laws. |  |  |  |  |  |
| 1. 9. | It is critical for government agencies to inculcate policies that can address and monitor online sales of medicines |  |  |  |  |  |

**Section D: Role of pharmacists**

| **S/N** | **Statement** | **SD** | **D** | **N** | **A** | **SA** |
| --- | --- | --- | --- | --- | --- | --- |
| 1. 1 | Pharmacists have the responsibility to differentiate genuine from counterfeit drugs |  |  |  |  |  |
| 1. 2 | Pharmacists have the responsibility report suspicious medicines to the regulatory officials |  |  |  |  |  |
| 1. 3 | It is the responsibility of Pharmacists to educate the public on the use Consumer Verification Strategies such as Mobile authentication service (MAS) to test the authenticity of medicines at the point-of-purchase. |  |  |  |  |  |
| 1. 4 | Pharmacists should at all times ensure that the purchase pharmaceuticals are from certified drug sources such as manufacturers and accredited distributors. |  |  |  |  |  |
| 1. 5 | Pharmacists have the responsibility to discourage patients from indulging in purchase of medicines from non-credible sources |  |  |  |  |  |
| 1. 6 | It is the responsibility of pharmacists to report suspected adverse drug reactions that may be indicative counterfeits. |  |  |  |  |  |
| 1. 7 | Pharmacists should undergo continuing development activities to enhance their knowledge on the detection of counterfeit medicines |  |  |  |  |  |
| 1. 8 | Pharmacists have the responsibility to always verify drug supply sources |  |  |  |  |  |
| 1. 9 | It is the responsibility of pharmacists to detect fake and counterfeit medicines |  |  |  |  |  |
| 1. 10 | Pharmacists can make substantial contribution to the national effort to combat counterfeit/fake drugs |  |  |  |  |  |

**What other measures do you think pharmacists can adopt to reduce the incidences of counterfeit drugs?**

**………………………………………………………………………………………………………………………………………………………………………………………………………………………………………..............................................................................................**

**Thank you for taking your time to complete this questionnaire.**
